# Does using a femoral nerve block for total knee replacement decrease postoperative delirium?

**DOI:** 10.1186/1471-2253-12-4

**Published:** 2012-03-10

**Authors:** Sakura Kinjo, Eunjung Lim, Laura P Sands, Kevin J Bozic, Jacqueline M Leung

**Affiliations:** 1 Department of Anesthesia and Perioperative Care, University of California, San Francisco, CA 94143-0648, USA; 2 School of Nursing, Purdue University, West Lafayette, IN 47907-2069, USA; 3 Department of Orthopaedic Surgery, University of California, San Francisco, CA 94143-0728, USA

## Abstract

**Background:**

The effect of peripheral nerve blocks on postoperative delirium in older patients has not been studied. Peripheral nerve blocks may reduce the incidence of postoperative opioid use and its side effects such as delirium via opioid-sparing effect.

**Methods:**

A prospective cohort study was conducted in patients who underwent total knee replacement. Baseline cognitive function was assessed using the Telephone Interview for Cognitive Status. Postoperative delirium was measured using the Confusion Assessment Method postoperatively. Incidence of postoperative delirium was compared in two postoperative management groups: femoral nerve block ± patient-controlled analgesia and patient-controlled analgesia only. In addition, pain levels (using numeric rating scales) and opioid use were compared in two groups.

**Results:**

85 patients were studied. The overall incidence of postoperative delirium either on postoperative day one or day two was 48.1%. Incidence of postoperative delirium in the femoral nerve block group was lower than patient controlled analgesia only group (25% vs. 61%, *P *= 0.002). However, there was no significant difference between the groups with respect to postoperative pain level or the amount of intravenous opioid use.

**Conclusions:**

Femoral nerve block reduces the incidence of postoperative delirium. These results suggest that a larger randomized control trial is necessary to confirm these preliminary findings.

## Background

Postoperative delirium is a common postoperative complication among older surgical patients, occurring in up to 60% of surgical patients after major surgery [1]. Postoperative delirium is associated with increased mortality and morbidity, greater medical expenses, prolonged hospital stays and poor functional status [2,3]. Although multiple risk factors for postoperative delirium have been proposed [2,4-7], the pathophysiology of postoperative delirium remains unclear. Previous work from our laboratory and the work from others suggest that postoperative pain is an important factor related to postoperative delirium [5,8]. However, the effects of postoperative pain management technique, especially the effects of peripheral nerve blocks on postoperative delirium, have not been investigated. Specifically, whether opioid-sparing techniques such as peripheral nerve blocks reduce postoperative opioid use and associated side effects such as delirium has not been studied.

Accordingly, we conducted a prospective nested cohort study in patients who underwent unilateral total knee replacement (TKR) to examine the effect of postoperative pain management on delirium. The aim of the present study was to compare the incidence of postoperative delirium between patients who had femoral nerve block for postoperative analgesia vs. those who did not. We chose this patient population because patients who undergo orthopedic joint replacement surgery are typically older and the incidence of postoperative delirium is high [8,9]. Moreover, joint replacements are relatively painful procedures and therefore aggressive postoperative pain management is critical to allow early physical therapy and mobilization.

## Methods

The study was approved by the Institutional Review Board of the University of California, San Francisco, and was part of a larger study examining the pathophysiology of postoperative delirium conducted from 2001-2011 at the University of California, San Francisco Medical Center. A subset of patients in this study (n = 31) was included in a previous paper evaluating the use of patient-controlled analgesia (PCA) in patients with postoperative delirium [10].

Before participating in the study, written informed consent was obtained from each patient. Inclusion criteria were patients who were ≥ 65 yrs of age who underwent unilateral TKR. Patients who were not able to speak English, not able to provide written informed consent, or those with moderate to severe dementia were excluded. We did not include patients who had an epidural catheter postoperatively, since most of our patients received anticoagulants for deep venous thrombosis prophylaxis, which have the potential to increase the occurrence of epidural hematoma. As a result, postoperative epidural analgesia has been rarely used in our institution in patients undergoing unilateral TKR.

The anesthesia and postoperative pain management methods were not controlled by the study. Two different postoperative pain management groups were compared in the study. Group 1: continuous femoral nerve block ± patient controlled analgesia (PCA), Group 2: PCA analgesia only. The patients in Group 1 received either general anesthesia with inhalational agents or spinal anesthesia with single shot femoral nerve block with local anesthetic (e.g., 30 ml of 0.5% ropivacaine) followed by continuous local anesthetic infusion in the femoral nerve catheter. The anesthesia team performed sensory and motor testing of femoral nerve block right before surgery. Group 2 received general or regional block (spinal or epidural) followed by intravenous PCA analgesia. The epidural catheter was discontinued in the Post Anesthesia Care Unit (PACU) before patient was transferred to the floor.

Continuous femoral nerve infusion and PCA were started in the PACU and both techniques were maintained typically for 1-2 days postoperatively. Local anesthetic (e.g., ropivacaine 0.2% at 8-12 mL/hour) was used for femoral nerve infusion and titrated to keep the pain scores < 5 per our institutional practice.

Hydromorphone was the standard opioid for postoperative pain management in our institution. If hydromorphone was not used, for example because of patient allergy or sensitivity, morphine sulfate or fentanyl was used. Morphine sulfate and fentanyl doses were converted to hydromorphone using the conversion formula: 5 mg of morphine sulfate = 1 mg of hydromorphone, 50 mcg of fentanyl = 1 mg of hydromorphone [11]. The dosages on the pain medications were not specifically modified based on age. Oral pain medication (e.g., hydrocodone with acetaminophen) was started as needed, typically on postoperative day (POD) 1, when the PCA was being discontinued.

### Preoperative patient assessment

The preoperative interview was conducted by a trained research assistant in the preoperative anesthesia clinic, typically less than 2 weeks prior to surgery. The preoperative interviewer collected health and demographic information associated with postoperative delirium, including age, gender, race, level of education, history of central nervous system (CNS) disorders, daily alcohol consumption, baseline cognitive status. The CNS disorders included delirium, dementia, depression, seizure and other CNS disorders. Information from the preoperative physical exam included American Society of Anesthesiologists (ASA) classification, and the use of preoperative opioids and benzodiazepine, and preoperative pain level were obtained.

Baseline cognitive status was obtained in person or over the phone preoperatively using the Telephone Interview for Cognitive Status (TICS) [12]. TICS is an 11-item test (with a maximum score of 41 points), which correlates well with the Mini-Mental State Examination and widely used for screening of dementia. Scores below 30 on the TICS are considered to indicate cognitive impairment [13].

Screening of depression was performed using 15-question Geriatric Depression Scale (GDS) [14] preoperatively. Our previous study demonstrates that patients with higher GDS scores (≥ 6), reflecting a larger number of symptoms of depression, are more likely to develop a longer duration of postoperative delirium.

In addition, patients were asked to rate their baseline pain levels using the 11-point numeric rating scales (NRS) from "no pain" to "worst possible pain" [15].

### Postoperative patient assessment

The same trained research assistant conducted the structured interviews, which occurred daily for 2 days postoperatively between approximately 9 a.m. and 12 p.m., in the patient's hospital room. Postoperative delirium was measured using the Confusion Assessment Method (CAM) [16]. Diagnosis of delirium requires the presence of: acute onset of mental changes and fluctuating course, inattention, and disorganized thinking and/or altered levels of consciousness as measured by the Confusion Assessment Method (CAM) Rating Scale. The CAM is a reliable and convenient tool for making a diagnosis of delirium and has high sensitivity (94-100%) and high specificity (90-94%) [16].

Pain levels at rest were measured in the same way as the preoperative evaluation, using the NRS. Information related to postoperative pain management (type of pain management and opioid usage) was obtained through the review of medical records.

Other potential variables (altered sleep-awake cycle and postoperative benzodiazepine use) for postoperative delirium were assessed. Altered sleep-wake cycle was defined as a change in the regular sleep pattern per subjective report by patient.

### Statistical analysis

First, if a patient completed at least 75% of items of the TICS and GDS, we substituted each item with item-mean value. Chi-square tests (Fisher's exact test if the cell count was less than 5) were used for the categorical variables. Mantel-Haenszel chi-square tests were used for the ordinal variables. Two sample t-tests were conducted for the continuous variables except opioid dose. Wilcoxon non-parametric test were performed to examine the association between postoperative pain management techniques and use of opioids. With bivariate correlates of *P *< 0.20, stepwise logistic regression was performed to determine the independent contribution of predictor variables on postoperative delirium. *P*-values < 0.05 were considered significant. All analyses were performed using SAS 9.2 (SAS Institute, Inc., Cary, NC).

## Results

Initially, we analyzed the group of patients who underwent TKR between 2001-2006 (total of 71 patients; 18 patients for femoral nerve block group and 53 patients for PCA only group). We found that there was a trend of lower incidence of postoperative delirium in the femoral nerve block group (33% vs. 58%, *P *= 0.06) with no differences in postoperative pain and opioid consumption. This preliminary result led to a further analysis and patients between 2007-2011 were added. The total number of patients who underwent non-cardiac surgery was 674, and 88 patients underwent TKR between 2001-2011. 2 patients who underwent bilateral TKR, 2 patients who had postoperative epidural infusion, and 1 patient who had femoral and sciatic nerve blocks were excluded. Thus, 85 patients met our criteria for this study; 31 patients had femoral nerve block ± PCA and 54 had PCA only. Patient characteristics and preoperative data are shown in Table 1. There were no significant differences in patient characteristics between the two groups except for ASA classification. There were more patients classified as ASA 3 or higher in the femoral nerve block group when compared with the PCA group. Table 2 shows the relationship between possible intra/postoperative risk factors for delirium and pain management groups. No significant differences were observed between the two groups with respect to patient's clinical data. Although there was a trend for less pain experienced on POD1 the change in pain levels (difference between preoperative pain and postoperative pain) was not significantly different. The amount of intravenous opioid consumption was significantly lower in the femoral nerve block group when compared with the PCA group 171.3 ± 160.8 mg vs. 259.9 ± 185.5 mg, *P *= 0.03). However, this significant difference in opioid use did not extend to the next postoperative day.

**Table 1:** Demographics and preoperative data

	Femoral Block ± PCA		PCA only		
	N1	Mean ± SD or N (%)	N2	Mean ± SD or N (%)	P*
Age	31	72.8 ± 5.8	54	74.5 ± 6.5	0.23
Gender	31		54		0.95
Female		18 (58%)		31 (57%)	
Male		13 (42%)		23 (43%)	
Race	31		54		0.84
White		23 (74%)		39 (72%)	
Non-white		8 (26%)		15 (28%)	
Education	30		52		0.17
Less than college		12 (40%)		29 (56%)	
College or above		18 (60%)		23 (44%)	
History of CNS disorders	31	18 (58%)	52	35 (67%)	0.40
Daily Alcohol	31		53		0.57
< 2 drinks		24 (77%)		38 (72%)	
≥ 2 drinks		7 (23%)		15 (28%)	
TICS Scores	28	33.1 ± 5.8	47	31.0 ± 3.8	0.09
(mean ± SD)					
GDS	30	3.1 ± 3.0	48	3.2 ± 2.7	0.93
(mean ± SD)					
ASA	31		54		0.01
1 and 2		8 (26%)		29 (54%)	
≥ 3		23 (74%)		25 (46%)	
Use of preoperative opioids	30	15 (50%)	53	17 (32%)	0.11
Use of preoperative benzodiazepine	30	5 (17%)	53	8 (15%)	0.85
Preoperative pain level	31	3.7 ± 3.2	52	2.5 ± 2.7	0.07

ASA = American Society of Anesthesiologists. CNS = central nervous system. GDS = geriatric depression scale. SD = standard deviation. TICS = Telephone Interview for Cognitive Status

For a continuous variable, the numbers in parenthesis are the range of the variable

*For *P*-value, i) one-way ANOVA was used for the continuous variables; ii) Chi-square test was used for the categorical variables. If the cell count was less than 5, Fisher's exact test was used; and iii) Mantel-Haenszel chi-square test was used for the ordinal variables

**Table 2:** Intraoperative and postoperative data

	Femoral Block ± PCA		PCA only		
	N1	Mean ± SD or N (%)	N2	Mean ± SD or N	P*
Anesthesia	31	14 (45%)	54	27 (50%)	0.67
General		14 (45%)		27 (50%)	
Regional		17 (55%)		27 (50%)	
Intraoperative fentanyl dose (mcg) ^†**^	31	171.3 ± 160.8	53	259.9 ± 185.5	0.03
Length of hospital stay (days)	29	5.7 ± 6.4	52	5.0 ± 1.9	0.58
Altered sleep-wake cycle on POD1	30	10 (33%)	51	25 (49%)	0.17
Pain at rest on POD 1	31	4.6 ± 3.0	53	4.5 ± 2.9	0.89
Change in pain level, preop and POD1	31	0.9 ± 3.2	52	1.9 ± 3.7	0.20
Benzodiazepine use on POD 1	31	2 (6%)	54	9 (17%)	0.18
Hydromorphone dose on POD1(mg)**	31	4.3 ± 4.6	54	5.9 ± 6.1	0.24
Delirium on POD 1 or POD2	28	7 (25%)	51	31 (61%)	0.002

POD = postoperative day. Preop = preoperative. SD = standard deviation For a continuous variable, the numbers in parenthesis are the range of the variable

^†^Intraoperative opioid: the amount of intravenous fentanyl administered intraoperatively

*For *P*-value, i) one-way ANOVA was used for the continuous variables; ii) Chi-square test was used for the categorical variables. If a cell count was less than 5, Fisher's exact test was used. **Wilcoxon non-parametric test was used

The overall incidence of delirium on either POD1 or POD2 was 48.1%. Table 2 shows that the rate of delirium was 25% for femoral nerve block group *vs*. 61% for the PCA only group (*P *= 0.002). Stepwise logistic regression analysis was performed using the bivariate correlates with *P*-value < 0.20 for postoperative delirium on POD1 or POD2. These variables were preoperative TICS scores and pain management (femoral nerve block ± PCA vs. PCA only) (Table 3). Pain management was significant for postoperative delirium (Odds Ratio =7.02, 95% Confidence Interval = (2.06, 23.97), *P *= 0.002) and preoperative TICS score was also significant (Odds Ratio = 0.87, 95% Confidence Interval = (0.77, 0.98), *P *= 0.03).

**Table 3:** Stepwise logistic regression on postoperative delirium (N = 71)

	Label	*P*- value	β	SE	Odds ratio	95% CI
TICS scores		0.03	-0.14	0.06	0.87	0.77-0.98
Postoperative pain management	PCA only vs. Femoral nerve block ± PCA	0.002	0.97	0.31	7.02	2.06-23.97

TICS = Telephone Interview for Cognitive Status. PCA = Patient controlled analgesia SE = standard error. CI = confidence interval. C-statistic = 0.75 (95% CI = 0.64-0.87)

We also investigated the impact of missing data on our results. Overall, 14 patients had incomplete delirium assessment or preoperative TICS score due to patients' refusal or medical condition. There was no significant difference between patients with missing and without missing in all variables. No catheter related complications were noted.

## Discussion

In this study, we found that the overall incidence of postoperative delirium to be 48.1%, which was consistent with that found in previous studies [9,17,18]. This relatively high incidence of delirium may be secondary to the older patient population being studied: the mean age of our cohort was 73.9 ± 6.3 years. In addition, the same trained research assistant evaluated patients' cognitive status daily, which may have increased the sensitivity of delirium.

Although postoperative delirium is prevalent and prognostically important, evidence-based preventive therapy is limited. Previous studies have investigated other prophylactic measures in preventing postoperative delirium using pharmacological (e.g., antipsychotics [19], anesthetic agents [20], and anticholinergics [21]) or non-pharmacological intervention (e.g., proactive geriatric consultation program [22]). Marcantonio et al. [22] studied patients with hip fracture in acute hospital settings. Patients were randomized in either proactive geriatric consultation group or standard care group. They found that a decrease in the rate of delirium in the geriatric consultation group, however the length of hospital stay was not significantly different in 2 groups. In contrast, results from pharmacological prevention of delirium are not conclusive. According to Larsen et al. [19], perioperative administration of antipsychotic agent, olanzapine decreased an incidence of postoperative delirium in patients underwent elective orthopedic surgery. Sieber et al. [20] reported that the use of light propofol sedation during spinal anesthesia decreased incidence of postoperative delirium by 50% compared with deep sedation. In contrast, a study by Gamberini and colleagues [21] found that administration of anti-cholinesterase inhibitor, rivastigmine did not decrease the rate of postoperative delirium in patients underwent elective cardiac surgery.

Our study is one of the first to investigate a relationship between the use of a femoral nerve block and postoperative delirium. In recent years, anesthesia and postoperative pain management techniques for TKR have changed substantially. Since anticoagulant drugs such as low molecular weight heparin are now commonly used to lower the risk of perioperative venous thromboembolism, femoral nerve block has become a more popular choice over epidural analgesia, as femoral nerve block does not carry the risk of epidural hematoma. Femoral nerve block has additional advantages including: 1) no opioid related side effects (e.g., nausea/vomiting, urinary retention, and pruritus); 2) the contralateral limb will not be affected; and 3) not associated with hypotension as may occur with an epidural.

Our study results showed that pain management was a predictor of postoperative delirium and also showed femoral nerve block reduced the rate of delirium. The current findings did show that the use of femoral nerve block reduced the amount of intraoperative opioid dose, but the opioid sparing effect did not appear to extend to the postoperative opioid. The reduced intraoperative opioid use is likely related to the bolus of local anesthetic (e.g., 30 ml of 0.5% ropivacaine) administered for femoral nerve block during the catheter placement. A single shot femoral nerve block with local anesthetic often lasts up to 12-24 hours. The lack of difference in postoperative opioid usage and pain level may be secondary to the location of the femoral nerve catheter. Capdevila et al. [23] reported that the course and location of continuous three-in-one block was totally unpredictable. The tip of the nerve catheter reached the lumber plexus in 23% of the patients, lay deep to the medial or lateral part of fascia iliaca in 33%, 37% respectively. They concluded that the quality of sensory and motor blockade depend on the location of the catheter tip. Therefore, femoral nerve catheter may not consistently produce sensory blockade of primary branches of lumber plexus (femoral, obturator and lateral femoral cutaneous). In addition, it spares the sciatic nerve.

### Potential limitations

We assessed patients' cognitive status and other clinical outcomes for 2 days postoperatively. If a patient had experienced cognitive changes later, the incident may have been missed. However, because the incidence of postoperative delirium is higher and surgical pain is intense the first few days after surgery, we believe that we have captured the most important time period in this study.

Pain assessment was conducted once daily during the patient interview. Because acute postoperative pain is dynamic and may fluctuate, we may not have evaluated the complex relationship between postoperative pain and delirium completely. However, from our experience, pain assessment once in the morning vs. twice a day assessments showed no significant difference in pain scores. In a separate pilot study of 20 patients evaluating the potential differences in pain scores between once a day vs. twice a day assessments, we found that only 2% of pain scores were significantly different between the two methods.

The initial analysis on patients between 2001-2006 showed a trend of lower incidence of postoperative delirium in the femoral nerve block group. This result led to the further analysis including more patients, and it became statistically significant for lower incidence of postoperative delirium and reduced intraoperative opioid consumption, but not statistically significant for the postoperative pain levels and opioid use. Our results should still be considered as pilot since sample size calculation shows that 61 subjects per group (total of 122) will be needed for a future trial, given a power of 80% and level of significance at 0.05. A prospective study that is adequately powered is indicated to confirm this preliminary finding.

Lastly, our study design was a nested cohort, therefore there could be a potential bias with this type of study. It was not randomized, although the two groups of patients were matched with respect to preoperative demographics except for ASA physical status, additional covariates that may have been important may have been missed and need to be considered in future prospective randomized trial.

## Conclusions

In summary, our study showed that femoral nerve block was associated with a lower incidence of postoperative delirium compared to the PCA only treatment. These results suggest pain management strategy should be considered in future trials of postoperative delirium.

## Competing interests

The authors declare that they have no competing interests.

## Authors' contributions

SK participated in the design of the study. EL performed the statistical analysis. LPS participated in the designed of the study and performed the statistical analysis. KJB participated in coordination of the study and helped to draft the manuscript. JML designed and oversaw the study. All authors read and approved final manuscript.

## Pre-publication history

The pre-publication history for this paper can be accessed here:

http://www.biomedcentral.com/1471-2253/12/4/prepub
